# Supplementary figures and images for: Full spectrum flow cytometry reveals mesenchymal heterogeneity in first trimester placentae and phenotypic convergence in culture, providing insight into the origins of placental mesenchymal stromal cells
Source: eLife. 2022 Aug 3;11:e76622. doi: 10.7554/eLife.76622 (PMC9371602; doi:10.7554/eLife.76622)

Table 3- source data 1

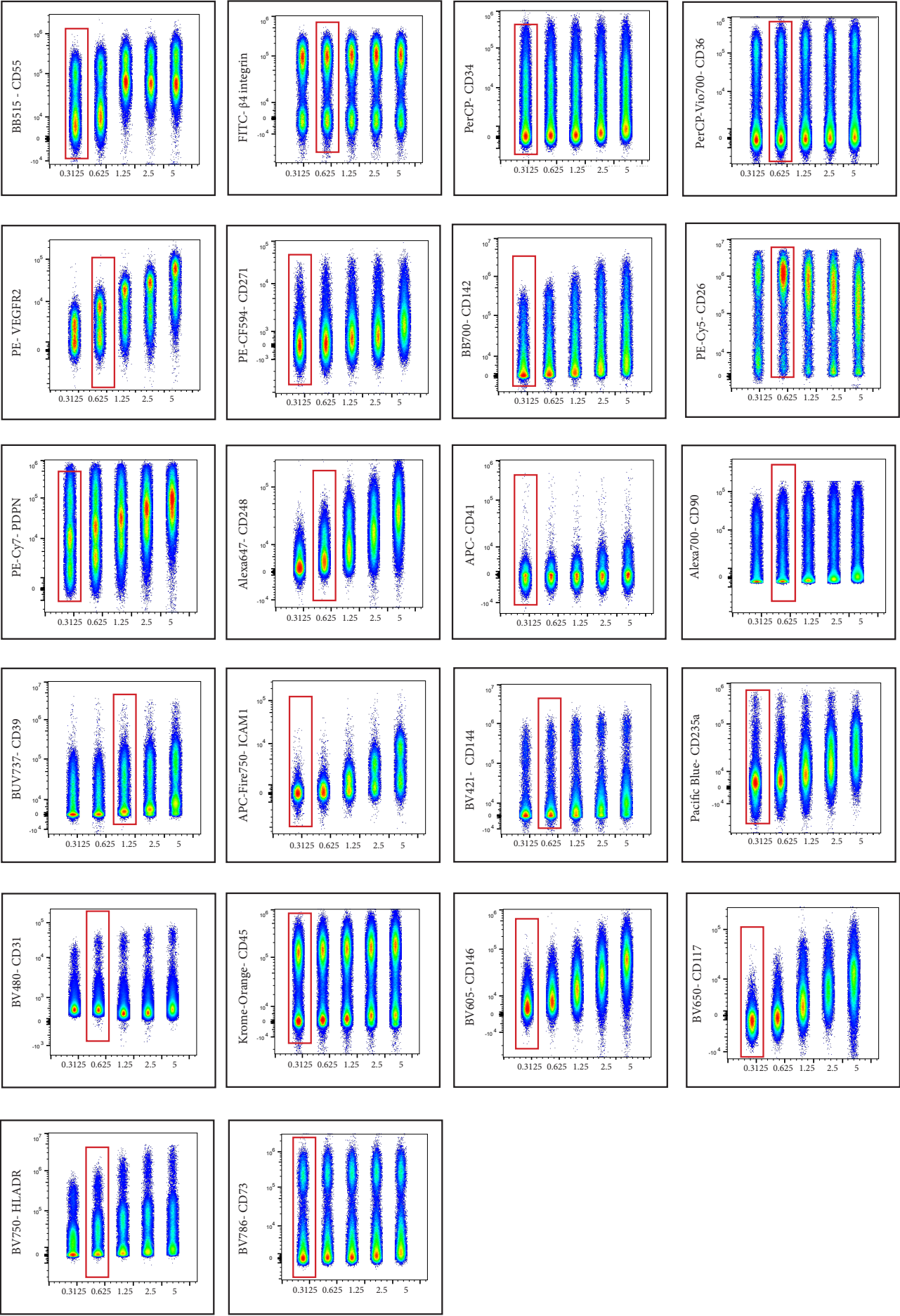

Supplement: Table 3—source data 1. [file elife-76622-table3-data1.pdf]

Table 3- source data 2

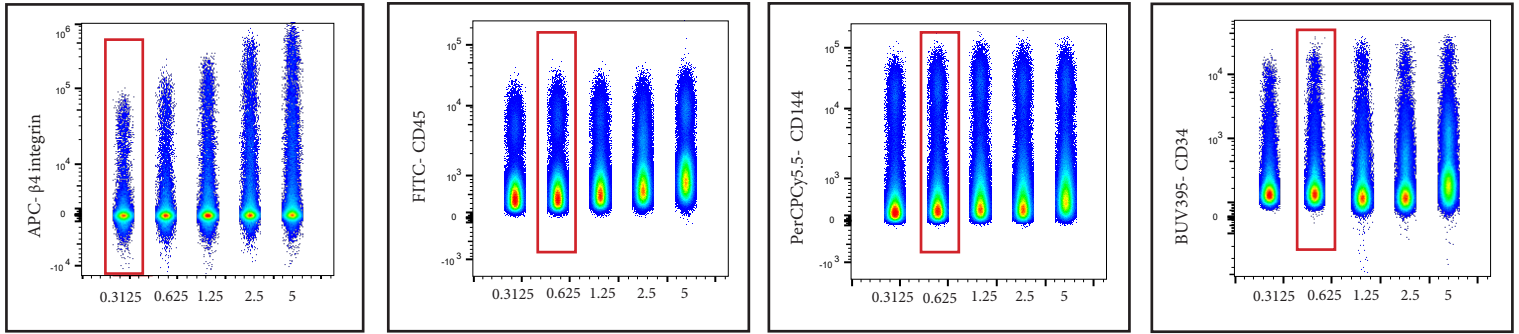

Supplement: Table 3—source data 2. [file elife-76622-table3-data2.pdf]

Table 3- source data 3

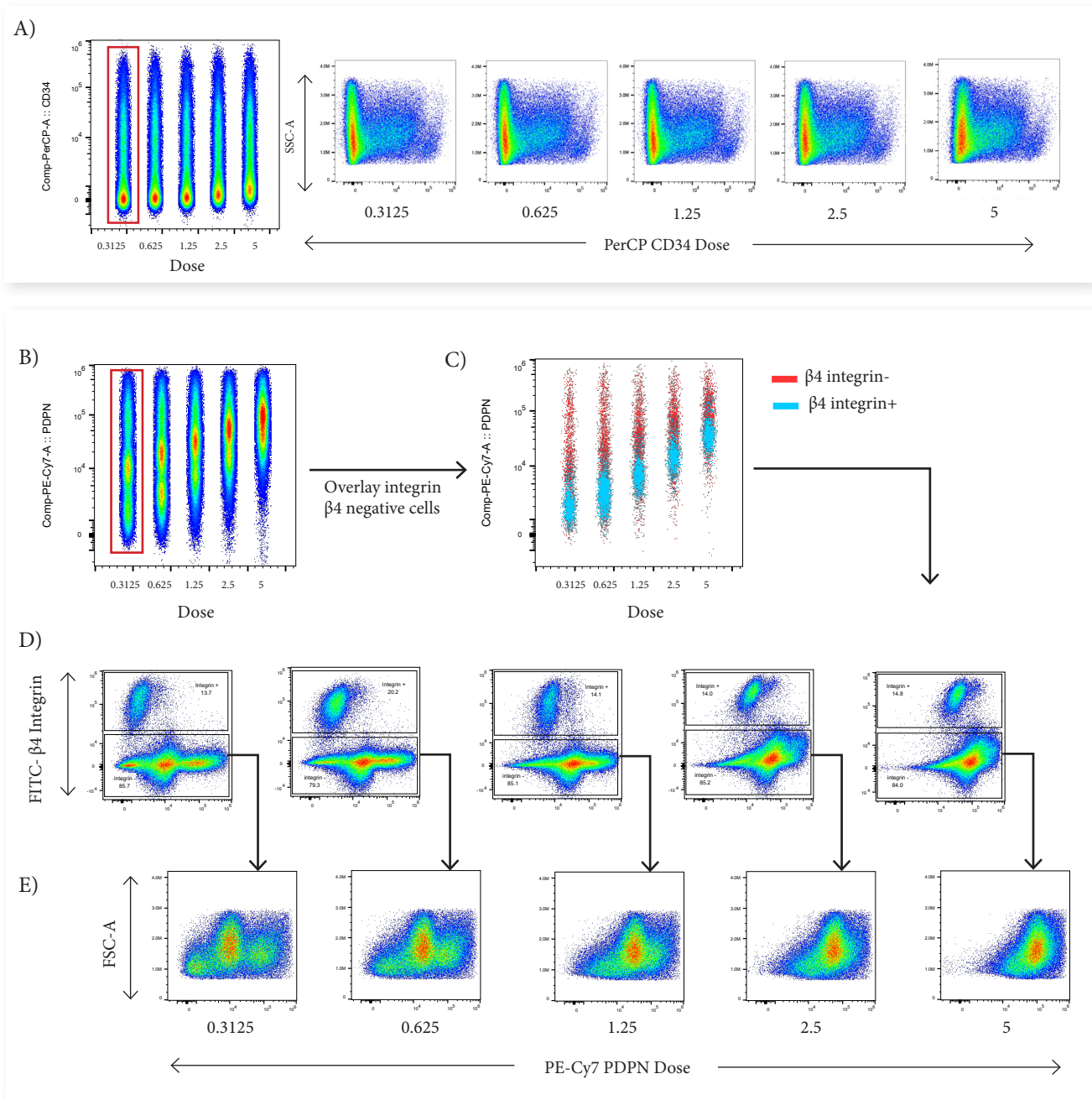

Supplement: Table 3—source data 3. — (B) β4 integrin+ cells were negative for podoplanin but demonstrated an unspecific shift in expression at higher doses. (C) Removal of β4 integrin+ improved detection of the optimal podoplanin dose. [file elife-76622-table3-data3.pdf]
